# Supplementary material for: Divergent Proteome Reactivity Influences Arm-Selective Activation of Pharmacological Endoplasmic Reticulum Proteostasis Regulators
Source: bioRxiv. 2023 Jan 17:2023.01.16.524237. Preprint. [Version 1] doi: 10.1101/2023.01.16.524237 (PMC9882204; doi:10.1101/2023.01.16.524237)
Supplement: 6 [file NIHPP2023.01.16.524237v1-supplement-6.pdf]

bioRxiv preprint doi: https://doi.org/10.1101/2023.01.16.524237; this version posted January 17, 2023. The copyright holder for this preprint (which was not certified by peer review) is the author/funder, who has granted bioRxiv a license to display the preprint in perpetuity. It is made available under aCC-BY 4.0 International license.

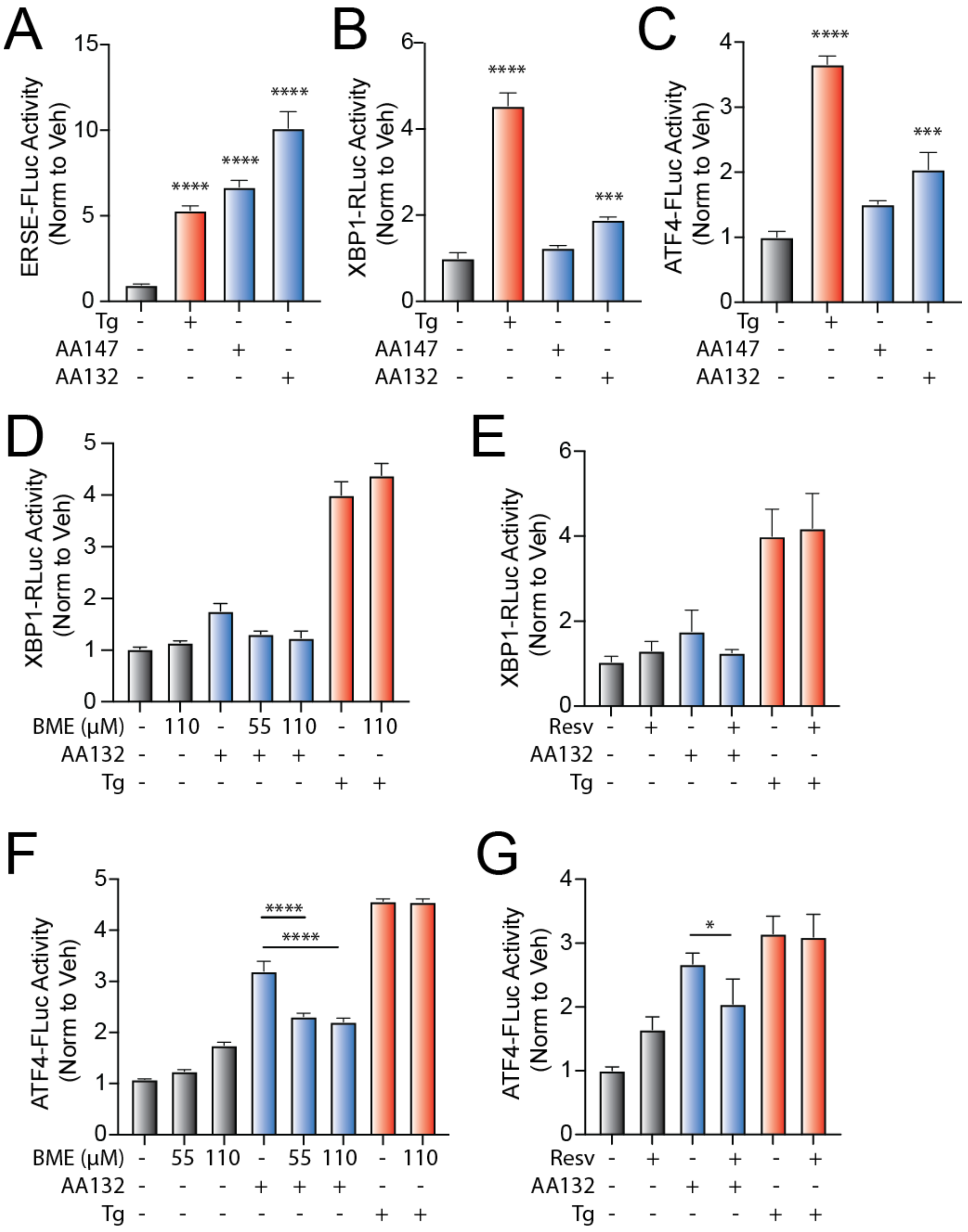

Supplementary Figure 1. AA132 activates ATF6 signaling pathways through a mechanism involving metabolic activation and covalent protein modification. A. Bar graph showing the activation of the ERSE.FLuc ATF6 reporter in HEK293T cells treated with Veh (0.1% DMSO), thapsigargin (Tg; 500 nM), AA147 (10 μM), or AA132 (10 μM) for 18 hr. Error bars show SEM for 6 independent experiments. ****$p < 0.0001$. **B.** Bar graph showing the activation of the XBP1s.RLuc IRE1 reporter in HEK293T cells treated with Veh (0.1% DMSO), Tg (500 nM), AA147 (10 μM), or AA132 (10 μM) for 18 hr. Error bars show SEM for 6 independent experiments. ***$p < 0.001$, ****$p < 0.0001$. **C.** Bar graph showing the activation of the ATF4.FLuc PERK reporter in HEK293T cells treated with Veh (0.1% DMSO), Tg (500 nM), AA147 (10 μM), or AA132 (10 μM) for 18 hr. Error bars show SEM for 6 independent experiments. ***$p < 0.001$, ****$p < 0.0001$. **D.** Bar graph showing the activation of the XBP1s.RLuc IRE1 reporter in HEK293T cells treated with AA132 (10 μM) or Tg (500 nM) in the presence or absence of β-mercaptoethanol (BME; 55 μM or 110 μM) for 18 hr. Error bars show SEM for 6 independent experiments**.** **E.** Bar graph showing the activation of the XBP1s.RLuc IRE1 reporter in HEK293T cells treated with AA132 (10 μM) or Tg (500 nM) in the presence or absence of resveratrol (2.5 μM) for 18 hr. Error bars show SEM for 6 independent experiments. **F.** Bar graph showing the activation of the ATF4.FLuc PERK reporter in HEK293T cells treated with AA132 (10 μM) or Tg (500 nM) in the presence or absence of β-mercaptoethanol (BME; 55 μM or 110 μM) for 18 hr. Error bars show SEM for 6 independent experiments. ****$p < 0.0001$ **G.** Bar graph showing the activation of the ATF4.FLuc PERK reporter in HEK293T cells treated with AA132 (10 μM) or Tg (500 nM) in the presence or absence of resveratrol (2.5 μM) for 18 hr. Error bars show SEM for 6 independent experiments. *$p < 0.05$.

bioRxiv preprint doi: https://doi.org/10.1101/2023.01.16.524237; this version posted January 17, 2023. The copyright holder for this preprint (which was not certified by peer review) is the author/funder, who has granted bioRxiv a license to display the preprint in perpetuity. It is made available under aCC-BY 4.0 International license.

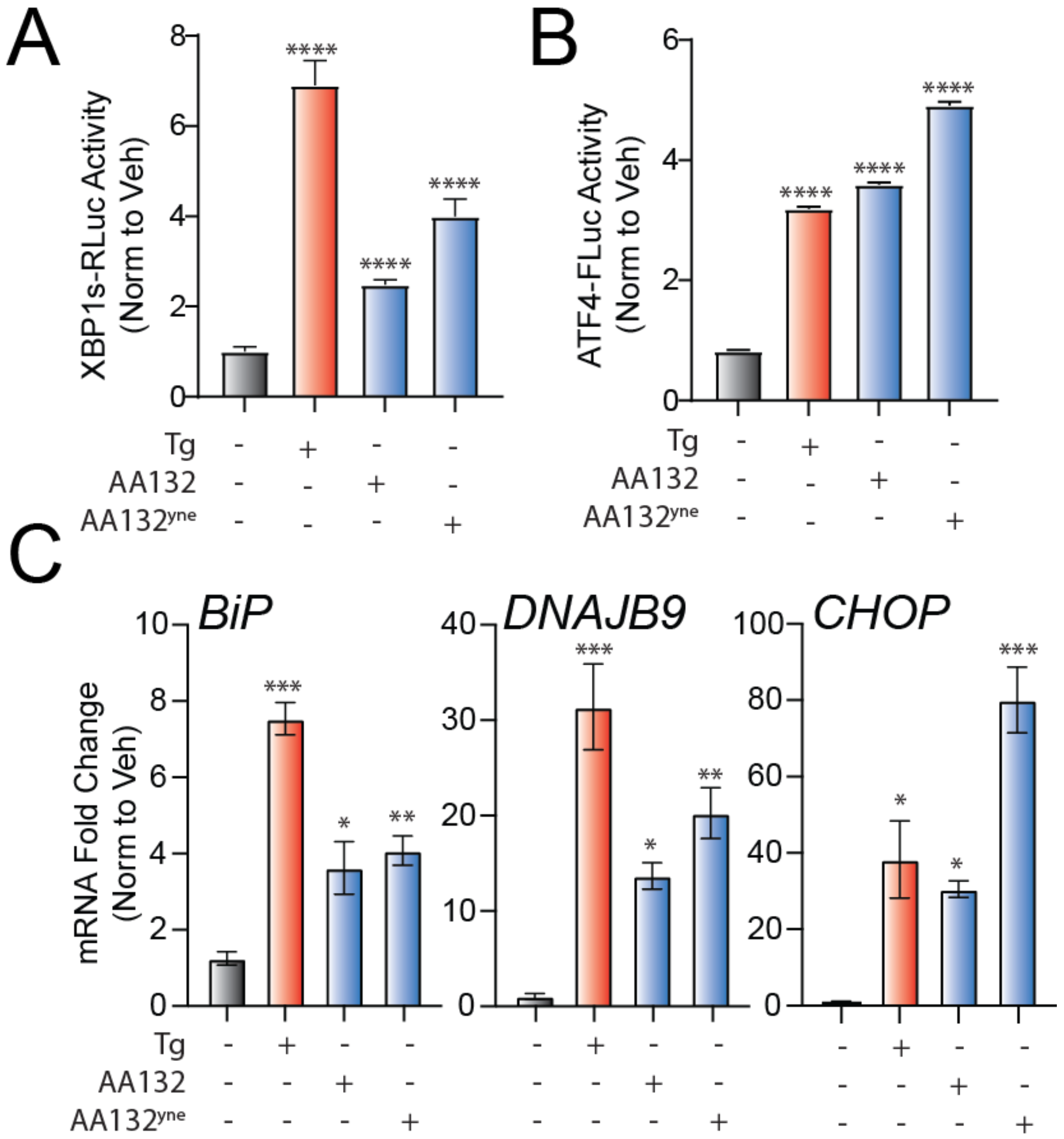

Supplementary Figure 2. Development of a Functional Affinity Enrichment Probe for AA132. A. Bar graph showing the activation of the XBP1s.RLuc IRE1 reporter in HEK293T cells treated with Veh (0.1% DMSO), thapsigargin (Tg; 500 nM), AA132 (10 µM), or AA132^{yne} (10 µM) for 18 hr. ****p<0.0001 **B.** Bar graph showing the activation of the ATF4.FLuc reporter in HEK293T cells treated with Veh (0.1% DMSO), Tg (500 nM), AA132(10 µM), or AA132^{yne} (10 µM) for 18 hr**.** ****p<0.0001 **C.** Graph showing qPCR of the ATF6 target gene *BiP*, PERK target gene *CHOP*, and XBP1s target gene *DNAJB9* in MEF cells treated for 6 h with the indicated compound (10 µM). N = 3 biological replicates. *p<0.05, **p<0.01, ***p<0.001.

bioRxiv preprint doi: https://doi.org/10.1101/2023.01.16.524237; this version posted January 17, 2023. The copyright holder for this preprint (which was not certified by peer review) is the author/funder, who has granted bioRxiv a license to display the preprint in perpetuity. It is made available under aCC-BY 4.0 International license.

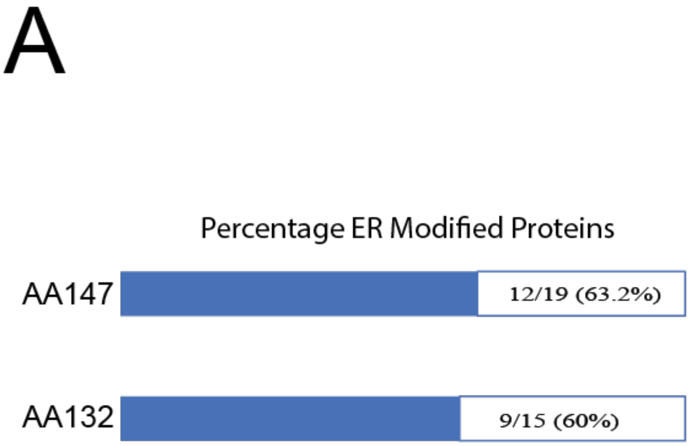

B

	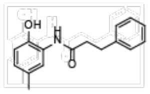 147	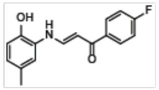 132
Molecular Weight(Formula)	C16H17NO2(255.31)	C16H14FNO2 (271.29)
Number Heavy Atoms	19	20
Fraction Csp3	0.19	0.06
# HBD/HBA	2,2	3,2
TPSA(Å^2)	49.33	49.33
Log P_{OW}	2.08	2.56

Supplementary Figure 3. AA132^{yne} Covalently Modifies ER PDIs. A. Graph showing proportion of AA147^{yne} and AA132^{yne} target proteins localized to the ER. **B.** Calculated physicochemical properties of AA147 and AA132**.** Values calculated using SwissADME (SwissADME.ch). TPSA = Total Polar Surface Area; HBD = Hydrogen Bond Donors; HBA = Hydrogen Bond Acceptors.

bioRxiv preprint doi: https://doi.org/10.1101/2023.01.16.524237; this version posted January 17, 2023. The copyright holder for this preprint (which was not certified by peer review) is the author/funder, who has granted bioRxiv a license to display the preprint in perpetuity. It is made available under aCC-BY 4.0 International license.

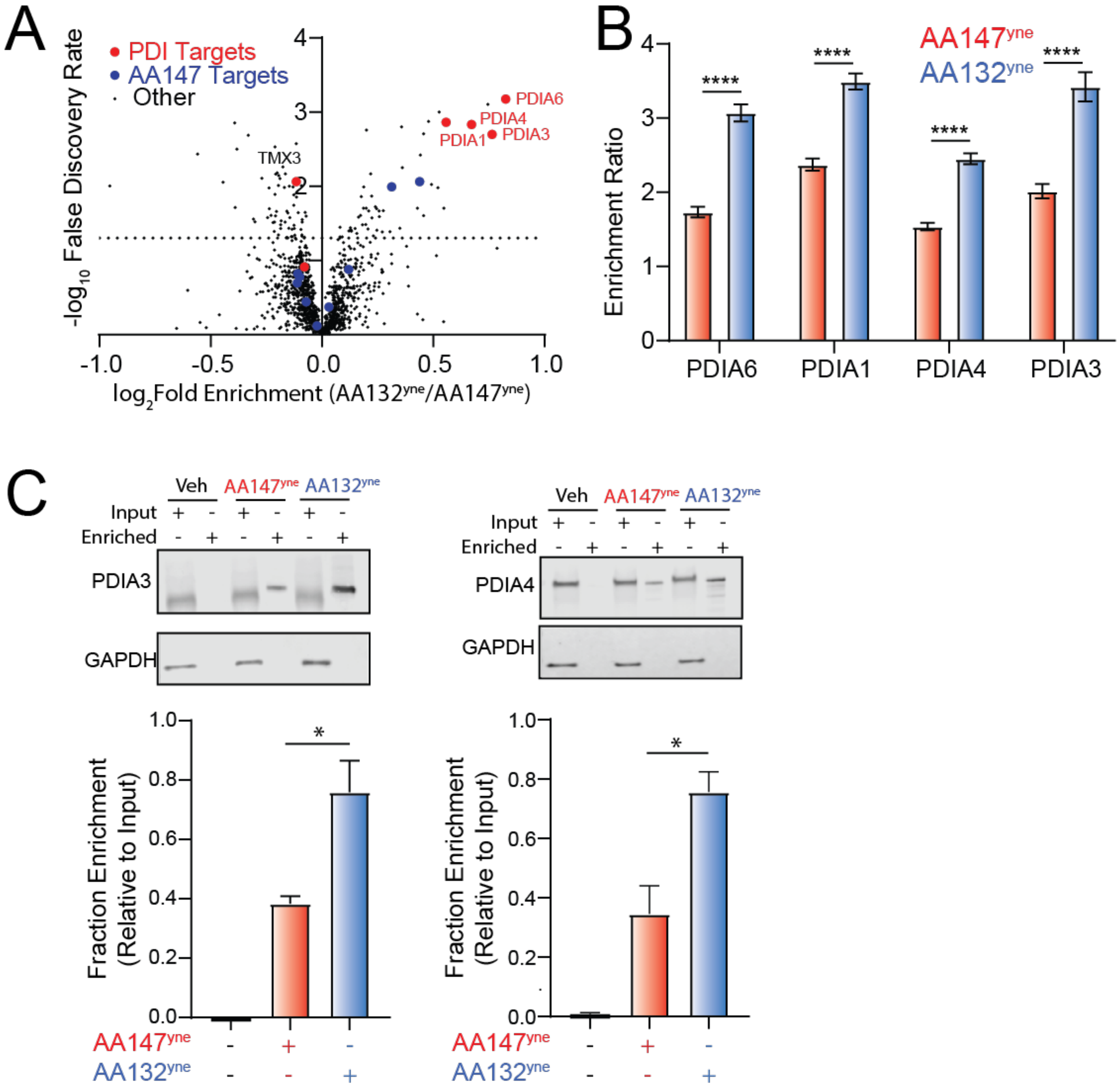

Supplementary Figure 4. AA132^{yne} Shows Higher PDI Labeling as Compared to AA147^{yne}. A. Volcano plot showing log_2 fold enrichment of AA132^{yne} labeled proteins relative to AA147^{yne} labeled proteins (x-axis) versus the –log FDR (y-axis) in HepG2 cells (10 µM, 6h). Proteins with GO annotation for PDI (GO: 0003756) labeled in red and additional previously defined AA147^{yne} targets labeled in blue. Data shown in **Table S2**. **B.** Bar graph of enrichment ratio of select PDIs by indicated the compound relative to DMSO from data shown in **Fig. S4A** (N = 4 biological replicates). ****p < 0.001 from multiple unpaired t-test. **C**. Representative immunoblot and quantification of PDIA3 and PDIA4 recovery in streptavidin enrichments from HEK293T cells treated with AA147^{yne} (10 µM; 6 h) or AA132^{yne} (10 µM; 6 h) and then conjugated to biotin. Fraction enrichment was calculated by dividing the signal in enriched samples by the input signal. *p < 0.05 from unpaired t test for N=3 replicates.

bioRxiv preprint doi: https://doi.org/10.1101/2023.01.16.524237; this version posted January 17, 2023. The copyright holder for this preprint (which was not certified by peer review) is the author/funder, who has granted bioRxiv a license to display the preprint in perpetuity. It is made available under aCC-BY 4.0 International license.

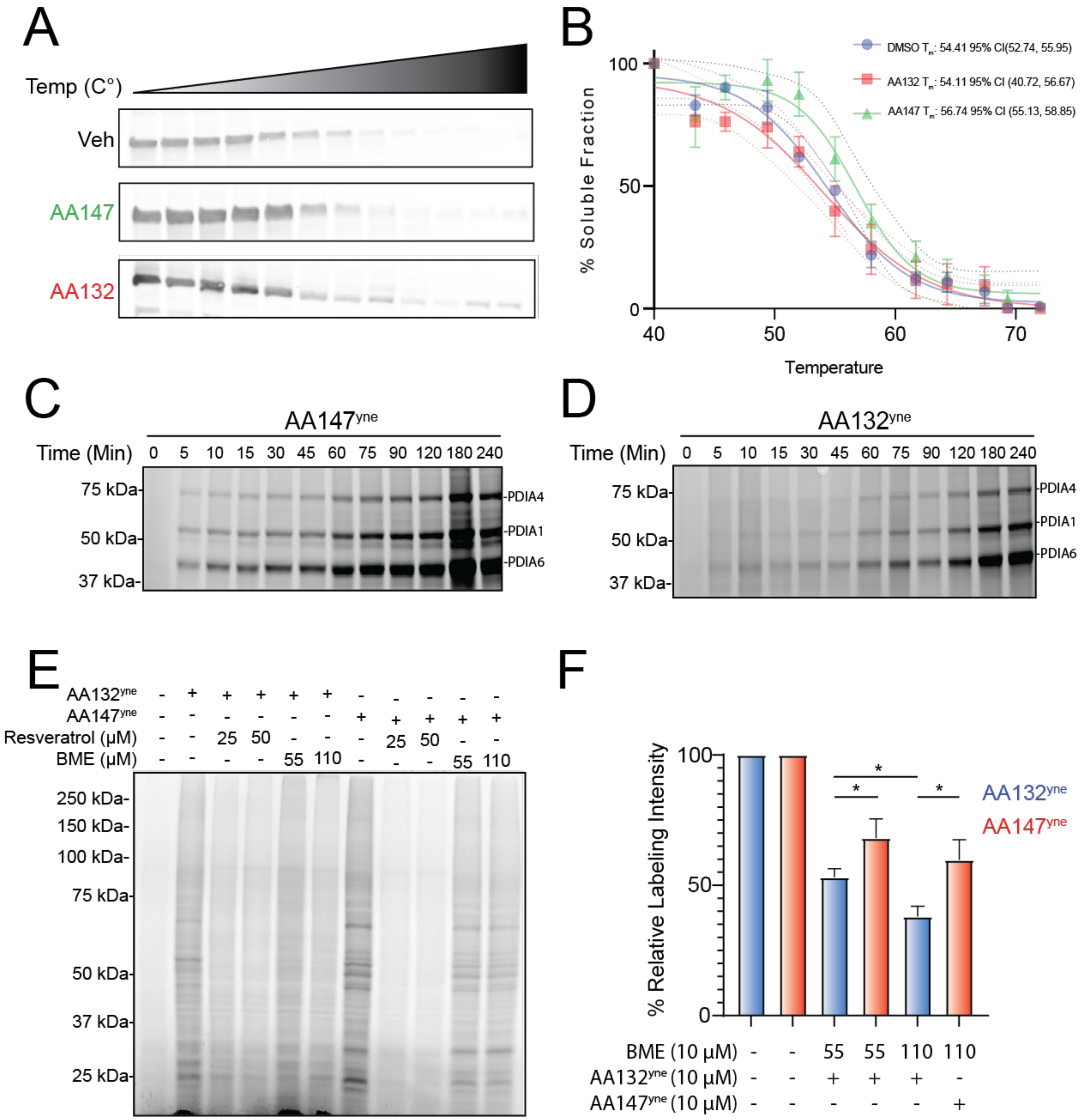

Supplementary Figure 5. AA132^{yne} Shows Slower Protein Labeling Kinetics as Compared to AA147^{yne}. A. Representative immunoblot of the soluble fraction of PDIA1 from heat-treated ALMC2 cells at the temperatures indicated (40-72°C) preincubated with listed compound (10 μM, 2h). **B.** Graph of percent soluble fraction (y-axis) versus temperature (x-axis) for data in **Fig S5A**. Fitted curves calculated using Boltzmann Sigmoidal Fit in Prism and plotted with 95% confidence intervals (dotted lines). T_m is temperature on calculated sigmoidal curve with 50% soluble PDIA1 fraction remaining. Error bars represent S.E.M (N = 3 biological replicates). **C.** Representative SDS-PAGE gel of Cy5-conjugated proteins from ALMC2 cells treated at indicated time point with AA147^{yne} (10 μM). **D.** Representative SDS-PAGE gel of Cy5-conjugated proteins from ALMC2 cells treated at indicated time point with AA132^{yne} (10 μM). **E.** Representative gel of AA132^{yne} and AA147^{yne} labeled proteins in HEK293T cells cotreated with indicated concentrations of β-mercaptoethanol or resveratrol for 4h. **F.** Quantification of **Fig S5E**. Error bars represent standard error of mean. *p* values calculated using two-sided Student T Test. Percent labeling calculated as lane intensity relative to cotreatment with vehicle (0.1% DPBS) *$p < 0.05$, **$p < 0.01$.

bioRxiv preprint doi: https://doi.org/10.1101/2023.01.16.524237; this version posted January 17, 2023. The copyright holder for this preprint (which was not certified by peer review) is the author/funder, who has granted bioRxiv a license to display the preprint in perpetuity. It is made available under aCC-BY 4.0 International license.

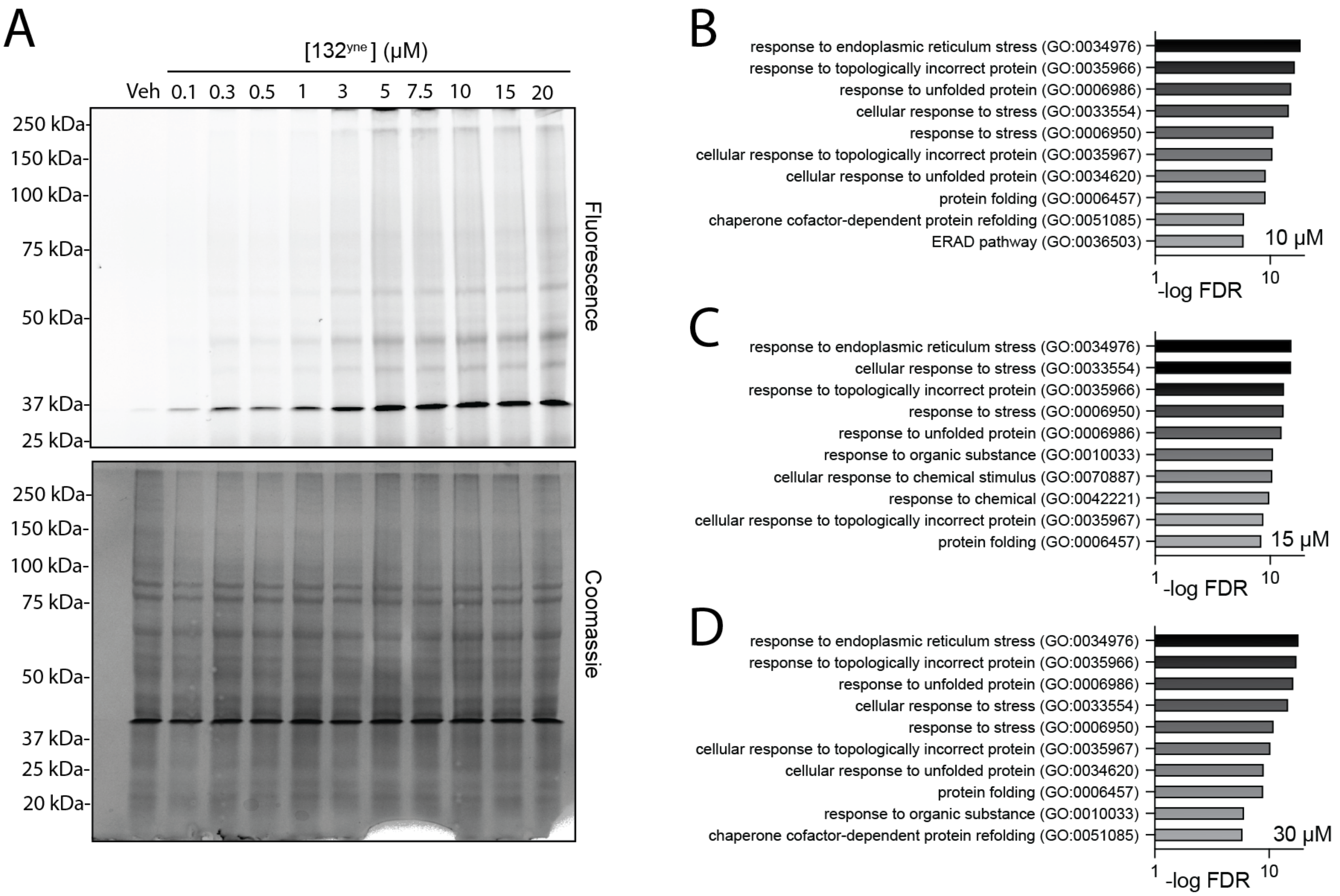

Supplementary Figure 6. AA132 Selectively Activates ATF6 Transcriptional Signaling at Lower Doses. A. Fluorescence and coomassie-stained SDS-PAGE of lysates prepared from HEK293T cells treated with the indicated concentration of AA132^{yne} (4 h) and then conjugated to azide-cyanine. **B-D**. Top-10 GO terms for significantly induced genes (fold change >1.3, p<0.05) identified by RNAseq in HEK293T cells treated with 10 μM (**A**), 15 μM (**B**), or 30 μM (**C**) AA132 for 6 h. RNAseq data is included in **Table S3** Full GO analysis is included in **Table S4**.

bioRxiv preprint doi: https://doi.org/10.1101/2023.01.16.524237; this version posted January 17, 2023. The copyright holder for this preprint (which was not certified by peer review) is the author/funder, who has granted bioRxiv a license to display the preprint in perpetuity. It is made available under aCC-BY 4.0 International license.

SUPPLEMENTARY TABLE LEGENDS

Table S1. Excel spreadsheet showing the enrichment and competition ratio for proteins identified as targets of AA132^{yne}. Related to **Fig. 3.**

Table S2. Excel spreadsheet showing fold enrichment for AA132^{yne}/AA147^{yne} of proteins identified in proteomics experiments performed in HEK293T or HepG2 cells. Related to **Fig. 4** and **Fig. S4**.

Table S3. Excel spreadsheet showing DESeq outputs for HEK293T cells treated with increasing doses of AA132 or AA147. Related to **Fig. 6**.

Table S4. Excel spreadsheet showing GO analysis for RNAseq data for HEK293T cells treated with the indicated concentration of AA132. Related to **Fig. 6**.

Table S5. Excel spreadsheet showing the expression of transcriptional targets of ATF6, IRE1/XBP1s and PERK signaling from RNAseq data of HEK293T cells treated with the indicated concentration of AA132 or AA147. Related to **Fig. 6**.
